# Effects of prenatal iron deficiency on neurological development and related disorders in offspring

**DOI:** 10.3389/fnut.2025.1637398

**Published:** 2025-09-05

**Authors:** Zejun Zhao, Yajun Shi, Miao Sun, Bin Wang

**Affiliations:** ^1^Department of Fetology, The First Affiliated Hospital of Soochow University, Suzhou, China; ^2^McKusick-Zhang Center for Genetic Medicine, State Key Laboratory for Complex Severe and Rare Diseases, Institute of Basic Medical Sciences Chinese Academy of Medical Sciences, School of Basic Medicine Peking Union Medical College, Beijing, China

**Keywords:** iron deficiency, pregnancy, fetal origins of adult disease, neurologic development, offspring, behavior

## Abstract

The fetal origins of adult disease hypothesis proposes that a variety of adverse stimuli during critical development stages can impair the structure and function of fetal organs, thereby increasing the risk of disease later in life. Iron affects fetal growth and development by facilitating oxygen and electron transport and by serving as a cofactor for enzymes that affect enzyme activity. Fetal iron deficiency (ID) can result from various factors during pregnancy, including inadequate maternal iron intake, maternal obesity, diabetes, smoking, prenatal stress, and prenatal alcohol exposure. These conditions disrupt fetal brain development and are associated with neurological disorders in offspring, such as cognitive impairment, anxiety, depression, schizophrenia, and autism. However, the mechanisms by which maternal iron deficiency leads to abnormal neurological development, as well as cognitive impairment and psychiatric disorders in the offspring, remain unknown. In this review, we summarize the causes of prenatal iron deficiency, the effects of iron deficiency on brain development and behavioral phenotypes, and the potential molecular mechanisms.

## 1 Introduction

Iron deficiency (ID) is a common nutritional deficiency worldwide, especially in women (1). Among pregnant women, the prevalence of ID is approximately 80% in developing countries and approximately 40% in developed countries (2). The global burden and inequality of ID continue to rise, which may be related to low utilization of public health intervention packages. Low socioeconomic status, low education levels, gender discrimination, religious beliefs, and frequency of antenatal care in countries with low Human Development Index (3–6). Iron–Folic Acid Supplementation (IFAS) is an effective strategy for preventing and managing prenatal iron deficiency anemia (IDA) during pregnancy. In Bangladesh, Ghana, the Philippines, and Northwest Ethiopia, the compliance with IFA intake among pregnant women is only 20%−50% (7–10). Even in developed countries such as Canada, there is a negative correlation between the socioeconomic status of pregnant women and the probability of a ferritin test (11). Currently, various healthcare systems lack effective policies for the detection and management of fetal iron deficiency (ID). The main indicators used to detect ID in pregnancy are ferritin, hemoglobin (Hb), and C-reactive protein (CRP) under inflammation (12). According to the US Preventive Services Task Force, there is insufficient evidence to support screening asymptomatic pregnant women for ID and IDA or treating them with iron supplements to prevent adverse maternal and infant health outcomes associated with IDA (13). The American College of Obstetricians and Gynecologists recommends screening hemoglobin levels for anemia rather than ID, universal supplementation with low-dose iron during pregnancy, and low-dose iron supplementation and prenatal vitamin therapy for pregnant women with IDA after determining the cause (13, 14). In Asia, most medical institutions use Hb concentration as a proxy for ID/IDA (14). They further diagnose ID using serum iron, total iron-binding capacity, and transferrin saturation (15). In line with WHO recommendation, pregnant women in Southeast Asia should take oral iron and folic acid supplements daily if the prevalence of anemia is exceeds 40%, or intermittently on a weekly basis if the prevalence is below 20% (14).

It is well-known that iron is crucial for maintaining the production of hemoglobin, which is the molecule that transports oxygen in the blood (16). In addition, iron is essential for maintaining cell development and metabolic function in the body, including DNA synthesis and repair, enzymatic activity, and mitochondrial function (17). The requirement for iron during pregnancy increases due to several factors: (1) the increased physiological plasma volume of pregnant women requires more iron to synthesize hemoglobin (18); (2) the fetus requires iron to synthesize endogenous reserves of iron as well as for its own oxygen transport and metabolism (19); and (3) the placenta, which is a metabolically active organ and a transporter between the maternal and fetal circuits, requires large amounts of iron (20).

**Figure 1:**
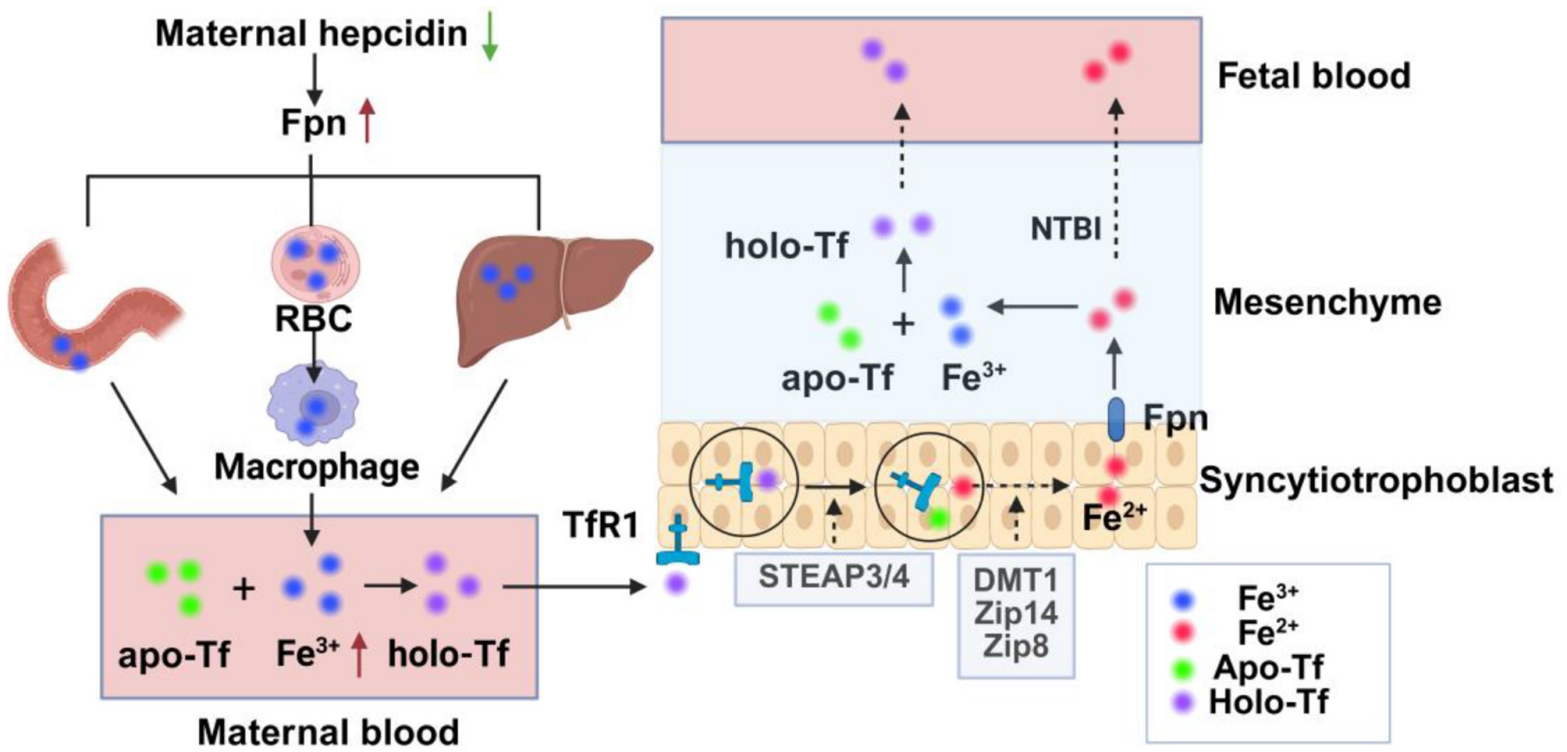
Schematic diagram of iron transport from mother to fetus. Maternal hepcidin is decreased during pregnancy, Fpn is increased, and iron flow into plasma is increased through increased intestinal iron absorption, macrophage iron recovery from aging red blood cells, and mobilization of ferritin in the liver. Fe^3+^ in plasma increases and binds to apo-Tf to form holo-Tf, which then binds to TfR1 on the apical membrane of placental trophoblastic cells to form the TFR1-transferrin complex, which is internalized by actin-coated vesicles. In vesicles, after the release of iron from transferrin, Fe^3+^ is reduced to Fe^2+^ by iron reductase and then transported from the vesicles to the interstitium by the iron transporters DMT1, Zip14, and Zip8. Fe^2+^ can be oxidized to Fe^3+^ after being transported by Fpn, which then combines with fetal Tf to form Holo-Tf and transported into the fetal blood, or directly into the fetal circulation in the form of NTBI. Fpn, ferroportin; RBC, red blood cell; STEAP3/4, 6-transmembrane epithelial antigen 3 and 4; Holo-Tf, holotransferrin, apo-Tf, iron-free transferrin; TfR1, transferrin receptor 1; DMT1, divalent metal transporter 1; Zip, Zrt/Irt-like protein; NTBI, non-transferrin-bound iron.

It is a priority to meet fetal iron needs in the case of mild maternal ID. However, the women with severe ID and exposure to adverse factors in pregnancy could cause fetal ID (19). Fetal ID affects fetal brain development, including hippocampal neuronal differentiation and synaptic plasticity and monoamine neurotransmitter metabolism (21). Neurodevelopmental abnormalities and mental health disorders, such as impairments in learning, memory, and emotion, occur in maternal ID offspring (22–24). In this review, we summarize the factors leading to prenatal fetal ID, the effects of prenatal ID on brain development and behavior of the offspring, the animal models of prenatal ID, and the possible mechanisms (Figure 1 and Table 1).

**Table 1:** Animal models used for maternal iron deficiency.

**Species**	**Age test**	**Prenatal iron deficiency conditions**	**Method**	**Offspring influence**	**References**
Rhesus monkeys	At birth	Give pregnant rhesus monkeys iron-deficient (10 mg Fe/kg) and iron-sufficient (100 mg Fe/kg) diet throughout pregnancy	Hematologic measures and bone marrow measures	Iron homeostasis was impaired in iron-deficient neonates.	(133)
Rhesus monkeys	First four months of life	Give pregnant rhesus monkeys iron-deficient (prenatal 10 mg Fe/kg or early postnatal 1.5 mg Fe/L formula) diet and iron-sufficient (prenatal 100 mg Fe/kg or early postnatal 12 mg Fe/L formula) diet throughout pregnancy	Motor and postural maturation, Novelty preference and eye–hand coordination, and grasp maturation	Weakened inhibitory response in iron deficiency group	(134)
Rhesus monkeys	Between 6 and 12 months of age	Give pregnant rhesus monkeys iron-deficient diet (prenatal 10 mg Fe/kg or early postnatal 1.5 mg Fe/L formula) diet and iron-sufficient (prenatal 100 mg Fe/kg or early postnatal 12 mg Fe/L formula) diet throughout pregnancy	Spatial maze, discrimination reversal (DR) task, concurrent object discrimination (COD) and delayed non-match to sample (DNMS) test	Weakened inhibitory response in iron deficiency group	(135)
Guinea pigs	P24 and P84	Give pregnant Guinea pigs iron-deficient diet (11.7 mg/kg) and iron-sufficient diet (114 mg/kg) from the beginning of pregnancy until postnatal day 9	Auditory brainstem response	Auditory brainstem response decreased in iron-deficient group.	(130)
Guinea pigs	P24	Give pregnant Guinea pigs iron-deficient diet (11.7 mg/kg) and iron-sufficient diet (114 mg/kg) from the beginning of pregnancy until postnatal day 9	ABR recording	Neural synchrony and auditory nerve conduction speed decreased in the iron-deficient group.	(131)
Guinea pigs	P24 and P40	Give pregnant Guinea pigs iron-deficient (10.1 mg/kg) diet and iron-sufficient diet (130 mg/kg) from the beginning of pregnancy until postnatal day 9	Open field test and Morris water maze test	Offspring anxiety increased in the iron deficiency group.	(132)
Rats	Between P25 and P67	Give pregnant rats iron-deficient diet (3 ppm) and iron-sufficient diet (45 ppm) 10 days before delivery	Fear conditioning test	The iron-deficient group had learning disabilities.	(127)
Rats	Between P32 and P39 for young rats and between P63 and P69 for adult rats	Give pregnant rats iron-deficient diet (3 ppm) and iron-sufficient diet (45 ppm) from 12 days gestation to 12 days after delivery	Eyeblink conditioning test	The learning ability of blinking conditioned reflex decreased in iron-deficient group.	(128)
Rats	P65	Give pregnant rats iron-deficient diet (3–6 mg/kg) and iron-sufficient (198 mg/kg) diet from 2 days gestation to 7 days after delivery	Eight-arm radial arm maze test	The learning ability of the offspring of iron deficiency group decreased.	(126)
Rats	Between P6 and P35	Give pregnant rats iron-deficient diet (2–6 ppm) and iron-sufficient (40 ppm) diet from 5 days gestation to weaning	Surface righting reflex, negative geotaxis reflex, sensorimotor function, and novel object recognition (NOR) task	Impaired myelination and behavior disorder in iron-deficient group	(81)
Rats	P15, P30, and P65	Give pregnant rats iron-deficient diet (3–6 ppm) and iron-sufficient (196 ppm) diet from 5 days gestation to weaning	Paired-pulse facilitation recordings and induction of long-term potentiation	Impaired synaptic plasticity in the iron-deficient group	(109)
Mice	3 months of age	Mice with hippocampal neuron-specific Slc11a2 knockout were obtained by mating Camk2a gene promoter-driven cre recombinase (cre) transgenic (Camk2a- cre) mice with Slc11a2 flox/flox mice	Morris water maze test	Disrupt hippocampal neuronal development and spatial memory behavior	(129)
Mice	2–4 months of age	Give pregnant mice iron-deficient (48 ppm) and iron-sufficient (96 ppm) diet from the beginning of pregnancy until postnatal day 10	Sucrose preference test, open field test, light–dark box test, forced swim test, and tail suspension test	Anxiety and depression-like behavior increased in the offspring of the iron-deficient group.	(23)

## 2 Iron homeostasis during pregnancy

**Figure 2:**
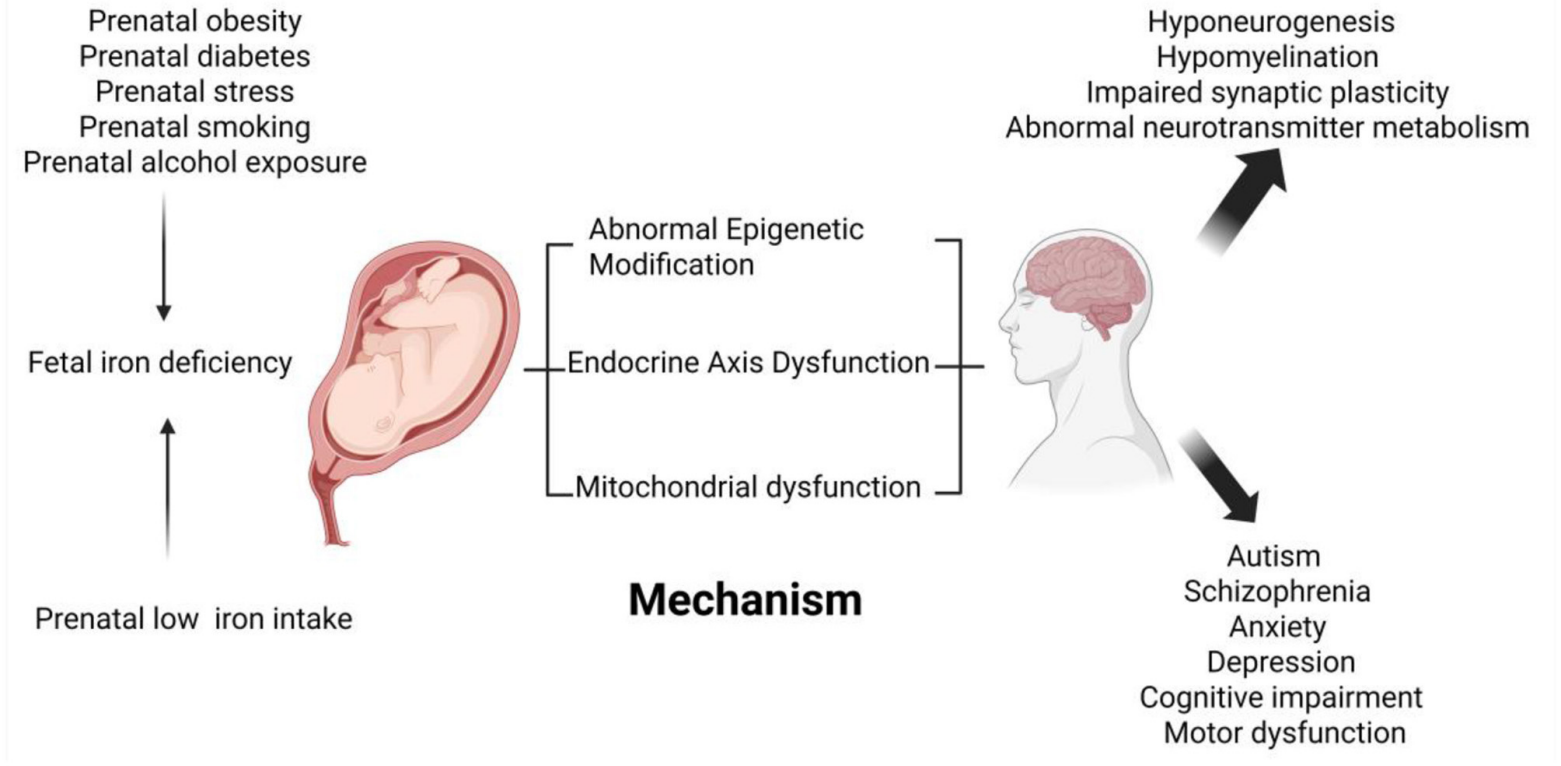
Schematic diagram of the effects of fetal iron deficiency on neurological development and related diseases. Low maternal iron intake during pregnancy, maternal obesity, maternal diabetes, prenatal stress, maternal smoking, and prenatal alcohol exposure have all been linked to fetal iron deficiency. Fetal iron deficiency affects fetal brain development, such as neurogenesis, emerging myelination, synaptic plasticity, and neurotransmitter metabolism, through epigenetics, endocrine axis dysfunction, and mitochondrial oxidative damage. Fetal iron deficiency is also associated with neurocognitive and mental health disorders in the offspring, such as depression and anxiety behaviors increased, impaired psychomotor development, learning and memory decline, autism, and schizophrenia.

Maternal physiological iron requirements rise significantly during pregnancy, with approximately 1 g of additional iron needed to maintain maternal iron homeostasis and to provide sufficient iron for fetal growth and development. Although maternal iron requirements are lower in the first trimester, they increase as the pregnancy progresses (25). Maternal hepcidin levels fall throughout the second and third trimesters of pregnancy, resulting in decreased binding and degradation of ferroportin (Fpn) (26, 27). Plasma iron is elevated through increased intestinal iron absorption, recycling of iron from senescent erythrocytes to macrophages, and mobilizing iron stores in the liver (26). In the interstitial fluid, iron ions bind to iron-free transferrin (apo-Tf) to form holo-transferrin (holo-Tf), which then binds to transferrin receptor 1 (TfR1) on the apical membrane of placental trophoblast cells to form the TfR1–transferrin complex. Upon binding, the TfR1–transferrin complex is internalized via clathrin-coated vesicles into an acidic environment (28). Then, the ferric iron (Fe^3+^) is separated from transferrin and reduced to ferrous iron (Fe^2+^) by iron reductases, such as the 6-transmembrane epithelial antigen of the prostate proteins 3 and 4 (STEAP3/4)(29). The specific pathway by which Fe^2+^ is transported from vesicles to the cytoplasm is unclear, and it may be related to iron transporters divalent metal transporter 1 (DMT1) and Zrt/Irt-like protein (ZIP) 8 (30, 31). Consequently, the TfR1–apolipoprotein complex returns to the membrane and is released (28). Fe^2+^ is exported from the syncytiotrophoblast by Fpn and oxidized to the Fe^3+^ by mammalian multicopper ferroxidases, such as ceruloplasmin, hephaestin, and zyklopen, which bind to fetal transferrin and are transported from endothelial cells to fetal circulation (Figure 2) (32–34).

When ID occurs during pregnancy, the expression levels of the molecules mediating placental iron uptake (TfR1) and output (Fpn) are altered. In mice, TfR1 increases, while Fpn decreases to maintain placental iron content in ID fetuses. In the placentas of pregnant women with mild ID, TfR1 expression is elevated, whereas Fpn remains unchanged. When severe ID is induced in human trophoblasts *in vitro*, TfR1 increases while Fpn decreases. The placenta cannot compensate for maternal ID to maintain fetal iron levels (35). In rats, fetuses exposed to prenatal ID can adaptively increase the expression of iron regulatory proteins (IRP-1 and IRP-2) and iron transport proteins (TfR and DMT1) in the hippocampus and the cerebral cortex to maintain brain iron requirements (36).

## 3 Causes of prenatal iron deficiency

### 3.1 Low maternal iron intake

Women with low iron intake, such as vegetarians or patients with gastrointestinal disorders, are at an increased risk of developing ID due to increased physiological iron requirements during pregnancy. Daily oral iron supplementation can decrease maternal anemia and full-term iron deficiency (37). When maternal ID is mild, iron is supplied preferentially to the fetus to ensure adequate fetal iron stores. However, when maternal ID is moderate or severe, fetal iron homeostasis is disrupted (2). Currently, it is believed that ID in pregnancy is defined as serum ferritin (SF) <30 μg/L (38). Fetal ID will occur when maternal ferritin concentration is less than 12 μg/L (39). Severe maternal ID is clinically manifested as iron deficiency anemia (IDA). When the maternal hemoglobin (Hb) concentration is <85 g/L, umbilical cord serum ferritin is <60 μg/L, indicating impaired fetal iron stores. When maternal Hb is <60 g/L, umbilical cord serum ferritin concentration is <30 μg/L, and umbilical cord Hb concentration is also decreased, indicating a progressive decline in umbilical cord ferritin levels as maternal anemia severity increases (40).

### 3.2 Maternal obesity

Maternal obesity and rapid weight gain are independent risk factors for fetal ID and are associated with elevated hepcidin levels (41–43). During pregnancy, high maternal body mass index (BMI) could induce maternal inflammatory responses, such as increased concentrations of interleukin-6 (IL-6) and C-reactive protein (CRP), which in turn lead to overexpression of hepcidin (44, 45). The increased number of macrophages in the placenta suggests that the inflammatory response in obese mothers extends to the uterus (46). Ultimately, fetal iron status is impaired. The released IL-6 forms a complex with the IL-6 receptor and glycoprotein 130 (gp130) to activate Janus kinase (JAK) (47). JAK phosphorylates tyrosine residues, which activates signal transducer and activator of transcription 3 (STAT3), and then enters the nucleus, binds to the hepcidin promoter, and induces hepcidin expression (48). In addition, maternal obesity increases the size of fat cells to produce more leptin, which induces hepcidin overexpression (49). In conclusion, obese women have a smaller decrease in hepcidin levels during pregnancy than non-obese women. Fpn located in intestinal cells, reticulocytes, and hepatocytes can bind more to hepcidin and be internalized and hydrolyzed by lysosomes, which can increase iron concentration in cells and reduce iron transport to plasma. However, some studies have shown that maternal obesity does not affect maternal and fetal iron status (50, 51), which may be related to the different degrees of maternal obesity and race.

### 3.3 Maternal diabetes

The offspring of diabetic mothers have abnormal iron distribution and decreased brain iron concentration, which may be associated with impaired iron transport (52, 53). In pregnant women with insulin-dependent diabetes, increased N-glycosylation of transferrin receptor (TfR) released from the placenta can reduce its binding capacity to transferrin (Tf), thereby reducing iron transport in the placenta (54). Then, decreased fetal iron reserve leads to increased expression of placental iron regulatory protein-1 (IRP-1), which binds to iron-responsive elements on the TfR mRNA and plays a stabilizing role in the upregulation of its expression (55, 56). However, Yang et al. (57) found that maternal iron transport to the fetus was reduced in gestational diabetes but was not associated with TfR expression.

### 3.4 Prenatal stress

Prenatal stress can lead to sex-specific fetal ID, which occurs predominantly in male fetuses and is associated with the fetal stress response system (58–63). Chronic stress can alter maternal expression of the acetylcholinesterase (AChE) gene, thereby converting the normal AChE-S splicing variant into an unstable AChE-R variant. On the one hand, the ratio of AChE-S to AChE-R can downregulate the expression of Fpn and metal ion transporters by modulating cholinergic pathway signaling via microglial α7 nicotinic acetylcholine receptors (α7nAChR) (64). On the other hand, elevated AChE-R is associated with chronic inflammation, which may lead to an increase in hepcidin (65). The above changes can reduce extracellular iron, thus affecting maternal and fetal iron homeostasis.

### 3.5 Maternal smoking

Fetal ID is associated with maternal smoking and is influenced by the frequency and number of days smoked. Iron stores might not be significantly impaired in pregnant women who smoke, but they are reduced in newborns. First, maternal smoking increases catecholamines in the maternal blood, which affects blood flow and vascular resistance in the placenta, as well as reduces blood nutrients and oxygen delivered to the fetus (66). Second, carbon monoxide in tobacco causes carboxyhemoglobinemia, which reduces the supply of hemoglobin to the fetus (67). Third, cyanide compounds contained in tobacco can exacerbate fetal hypoxia by impairing fetal oxidative mechanisms (67). Fourth, maternal smoking is positively associated with increased fetal lead concentration, which may contribute to hypoxia by interfering with hemoglobin synthesis and reducing the number of red blood cells (68). Conversely, a study showed that fetal iron homeostasis was not affected when women were exposed to smokeless tobacco during pregnancy (69).

### 3.6 Prenatal alcohol exposure

In pregnant women, prenatal alcohol exposure (PAE) increases maternal ferritin levels and decreases maternal hemoglobin-to-log (ferritin) ratio (70). In the fetus, PAE can decrease iron concentration and iron utilization in the brain (71). This may be related to elevated maternal and fetal hepcidin and the transfer of iron from the fetal brain and erythrocytes to the liver for storage. PAE can increase maternal and fetal inflammatory cytokines, such as IL-6, and activate hepcidin transcription, which impairs fetal iron homeostasis through the JAK/STAT signaling pathway (72). Due to PAE, serum ferritin (SF), Tf, and TfR cannot be upregulated in time, while fetal brain iron level drops (73). In addition, PAE upregulates the expression of the IL-1β gene in the placenta, which increases iron storage via promoting iron uptake into macrophages, the destruction of erythrocytes, and ferritin biosynthesis, as well as blocks the transport of iron in cells by inhibiting FPN-1 (74).

### 3.7 Gene–environment interactions

Gene–diet interactions and diseases indicate that genetic variations can also influence iron absorption and utilization. Haptoglobin (Hp) prevents oxidative damage mediated by free heme iron by removing it from cells (75). Hp gene polymorphisms constitute three main phenotypes: Hp 1-1, Hp 2-1, and Hp 2-2 (76). During mid-to-late stages of pregnancy, Hp phenotypes may increase susceptibility to ID in pregnant women. Pregnant women carrying the Hp 1 alleles may have increased susceptibility to ID if they do not have sufficient dietary iron intake or use prenatal supplements related to erythropoiesis. Additionally, obese women carrying the Hp 2-2 phenotype may have an increased risk of developing functional ID (77).

## 4 Consequences of iron deficiency on the nervous system

In fetal iron homeostasis, iron allocation is prioritized for red blood cells, rendering the brain susceptible to ID-mediated impairment even when hemoglobin levels remain within the normal ranges (21). Adequate iron during fetal brain development is necessary for neurogenesis, myelination, synaptic plasticity, and energy metabolism in neuronal and glial cells. Different durations and degrees of ID and the developmental stage at which ID occurs have various effects on brain development and function (21).

### 4.1 Reduced neurogenesis

Prenatal ID is associated with the inhibition of neurogenesis in the hippocampus of offspring mice and a reduction in the number of pyramidal cells and granule cells. The occurrence of ID at different stages of pregnancy may contribute to selectively change the volume of different parts of the fetal hippocampus and affect corresponding memory function. The critical time for susceptibility of the CA1 region of the hippocampus to ID is prenatal, and the dentate gyrus region of the hippocampus is susceptible to ID both prenatally and postnatally. Prenatal ID induces reduced neurogenesis and altered hippocampal volumes in the offspring, which may be associated with reduced brain-derived neurotrophic factor (BDNF) signaling (78).

### 4.2 Inhibition of myelin regeneration

ID is associated with myelin degeneration in both human studies and animal models. In human studies, the latency of auditory brainstem potentials as indirect markers of myelination is prolonged in infants with ID (79). In a rat model, severe ID may lead to persistent hypomyelination, the production of immature astrocytes, and increased pericyte permeability in offspring exposed to a maternal iron-deficient diet (80). Delayed myelination in specific parts of the brain is associated with behavioral disorders in rats (81). Oligodendrocyte progenitor cells (OPCs) and mature myelin oligodendrocytes are rich in iron and play an important role in myelination. In the brain, insufficient iron supply affects enzyme synthesis, which further affects the proliferation and differentiation of OPCs and myelin synthesis (82, 83). On the one hand, TfR expression on OPCs peaks during oligodendrocyte maturation and declines in mature myelinating cells to maintain iron homeostasis and development (84–86). Due to ID in the brain, the binding of apo-Tf produced by oligodendrocytes and epithelial choroid plexus cells to iron is decreased. This impairs holo-Tf formation, which is required for high-affinity binding to TfR, ultimately leading to a decreased iron uptake by OPCs (87). The effects of apo-Tf on oligodendrocyte maturation and myelination may be mediated by the following signaling pathways: (1) Apo-Tf injection improves oligodendrocyte maturation and myelination by the Notch signaling pathway, which participates in focal demyelination and regeneration by increasing the F3/contact protein levels and Hes5 expression (88, 89). (2) The Fyn/MEK/ERK and PI3K/Akt pathways are also active post apo-Tf treatment (90, 91). Iron-related pathways (e.g., Fyn/MEK/ERK, PI3K/Akt, Notch) are closely related to neurological diseases, such as cognitive impairment and schizophrenia (92–94). On the other hand, ferritin in oligodendrocytes consists of an equal combination of heavy chain (Fth) and light chain (Ftl) (87). As an antioxidant protein, Fth may prevent the formation of reactive oxygen species, but it also increases cytoplasmic iron levels and oxidative stress (87). In oligodendrocyte-specific Fth1 KO mice, knocking out Fth in oligodendrocytes leads to neuronal loss and oxidative damage, thus affecting myelination (95).

Under ID, the increased proliferation of astrocytes and the decreased expression of glial fibrillary acidic protein (GFAP) and connexin 43 (CX43) suggest that astrocyte maturation is impeded (96). Astrocytes can inhibit remyelination by secreting cytotoxic factors and conversely promote myelin repair by secreting trophic factors, such as tumor necrosis factor-α (TNF-α) and cytokines interleukin-1β (IL-1β) (97–99). Insulin-like growth factor 1 (IGF-1) in response to TNF-α and fibroblast growth factor 2 (FGF-2) in response to IL-1β are important for myelination (97, 100). Astrocytes have high expression of iron influx proteins and iron efflux proteins and, thus can safely uptake and recycle iron in the brain during demyelination (101). Iron distribution in astrocytes is critical for the remyelination process. When the iron efflux transporter Fpn is knocked out in astrocytes, there is a decrease in the proliferation of OPCs and a decrease in the expression of IL-1β and IGF-1, which are associated with decreased remyelination (102). When multi-copper ferroxidases are knocked out in astrocytes, iron efflux is impaired and free radical production is increased, which ultimately drives myelin damage (103).

### 4.3 Impaired synaptic plasticity

Synaptic plasticity is defined as the ability of synapses to change their structure, connectivity, and function in response to internal or external stimuli (104). In newborns, a previous study has shown that low maternal iron intake accelerates the decline of fractional anisotropy (FA) values in gray matter, indicating reduced synaptic formation and dendritic arborization in offspring (105). In rodents, fetal ID regionally affects dendrite morphology and branching before adulthood, despite subsequent iron supplementation in the brain (106–108). ID reduces the basal dendrite length of pyramidal neurons in the hippocampus without affecting branch complexity and increases the proximal branches of apical dendrites without affecting total length. In contrast, both apical and basal dendrite branch complexity are reduced in cortical neurons, but total length remained unchanged (107). The decreased long-term potentiation (LTP) indicates abnormal synaptic plasticity in fetal iron-deficient mice (109). ID leads to the reduction of four synaptic proteins: CaMKIIα, PSD-95, Fkbp1a, and Vamp1 (110). During repeated stimulation, iron deficiency can change synaptic plasticity by keeping the content of synaptic vesicles constant but reducing their release, and iron supplementation partially reverses this (111).

### 4.4 Abnormal neurotransmitter metabolism

Iron is involved in the synthesis of neurotransmitters as a cofactor of enzymes such as tyrosine hydroxylase and tryptophan hydroxylase (112). The striatum, one of the basal ganglia of the brain, delivers dopamine-rich substances to the prefrontal cortex and is involved in cognitive and motor functions (8). Altered dopamine function has been associated with an increased risk of schizophrenia in adult offspring of maternal ID (113). In the striatum, dopamine concentration is high due to high iron concentration (114). When ID occurs, cellular uptake of dopamine is reduced because the density and function of dopamine transporters as well as dopamine receptors are reduced in the caudate-putamen (115, 116). Injection of physiological iron concentrations into the ventral midbrain (VMB) alleviates ID-induced decrease in dopamine concentration in the striatum (117). Thy-1 is a cell adhesion molecule that regulates the release of neurotransmitter vesicles, and the fact that ID leads to a decrease in Thy-1 provides a new explanation for impaired dopaminergic transmission in the brain (118). Changes in local monoamine metabolism across various brain regions are sensitive, proportional to the degree of ID, and occur prior to the severe decrease in brain iron concentration (114, 119). Prenatal ID is associated with impaired monoamine metabolism in the offspring's brain, leading to abnormalities in learning and memory functions. These changes cannot be treated by postpartum iron supplementation (120). In addition, ID can also alter the density of serotonin transporters and norepinephrine transporters, which is more pronounced in male offspring (121).

## 5 Animal models used for iron deficiency

To better understand the effects of iron deficiency during pregnancy on brain development and behavioral phenotypes in animal offspring and the possible mechanisms involved, multiple animal models of ID in pregnancy have been established, as shown in Table 1.

In terms of pregnancy physiology, mice and rats have shorter gestation periods and multiple pregnancies, with fetuses being born with underdeveloped organs (122, 123). Regarding endocrinology, the entire pregnancy period is highly dependent on ovarian progesterone production to maintain pregnancy (123). With regard to the structure and efficiency of the placenta, there are uterine endothelial cells, maternal capillary endothelial cells, trophoblast cells, and fetal capillary endothelial cells between maternal blood and fetal tissue, resulting in low efficiency of material exchange (122). Guinea pigs have a longer gestation period than rats and mice, produce fewer offspring, and experience a rapid phase of brain development at birth (123, 124). Guinea pig placenta is discoid, labyrinthine, and haemomonochorial, resulting in higher efficiency of material exchange across the placenta than mice and rats (122). Rhesus monkeys are also similar to humans because they experience single-offspring pregnancies, have similar hematological changes during pregnancy (125), and share characteristics with humans in terms of placental transport, relative fetal growth, and regional brain development (21).

The animal model of ID during pregnancy has been established by restricting dietary iron intake of pregnant mothers to study its effect on adverse outcomes in offspring. Rats and mice are the most common animal models. The rodent brain at 10 days of gestation is considered equivalent to the human brain at full-term birth. Therefore, most rodent models of maternal ID are given an ID diet from pregnancy to about postnatal day 7, followed by which they are given an iron-sufficient diet (21). The pregnant rats are given an iron-deficient diet until after delivery, which decreases offspring's brain iron concentration, delays myelination, and impairs synaptic plasticity (81, 109, 126). Moreover, the offspring of rats exposed to maternal ID have impaired cognitive development, such as poor hippocampus-mediated spatial recognition learning and hippocampus-dependent trace fear conditioning and eyeblink conditioning (126–128). In addition, there are behavioral impairments in tests such as surface correction reflex and novel object recognition task (81). In the maternal iron-deficient mouse model, decreased iron level in the brain of the offspring is associated with anxiety and depression in adulthood (23). The mouse model with hippocampal neuron-specific knockout of Slc11a2, a gene responsible for iron uptake, showed that reduced iron content impaired the memory function by affecting the hippocampal neurodevelopment, including energy metabolism and dendrite morphology (129). When ID occurs in pregnant guinea pigs, the hearing function of offspring is impaired by affecting neural synchronization and auditory nerve conduction velocity (130, 131). Offspring of prenatal iron-deficient guinea pigs have increased locomotor activity, suggesting increased nervousness due to anxiety (132). Rhesus monkeys in the experimental group were fed a low iron diet (10 mg Fe/g) from 28 to 30 days of gestation until delivery, while controls were fed an iron-rich diet (100 mg Fe/g) (133). Newborns of pregnant Rhesus monkeys with ID were born with reduced hemoglobin, volume, and number of red blood cells but without neurobehavioral abnormalities (133). Other studies have shown that the offspring of prenatal ID in rhesus monkeys have reduced spontaneous activity in a new environment and behavioral disorders such as reduced inhibitory responses (134, 135). Moreover, the offspring of prenatal ID in rhesus monkey showed more active exploration in a new environment and in the manipulation of new objects than the control group, suggesting the presence of impulsive behavior syndrome (134).

## 6 Abnormal neurological behaviors

### 6.1 Motor function

Psychomotor development mainly encompasses gross and fine motor skills, and if impaired, it can affect an individual's cognitive and emotional development (136). The gross motor scores consist of three parts: reflexes, locomotion, and stationary subscales. Reflexes are automatic responses to environmental changes, such as the righting reflex. The assessment of locomotion is based on the ability to move from one place to another. In addition, the assessment of stationary is based on the ability to control the center of gravity and maintain balance (137). Fine movement mainly focuses on the use of body muscles to complete specific actions, such as finger movements and hand–eye coordination (138). During pregnancy, the offspring of anemic mothers with low hemoglobin concentration have slightly low gross and fine motor scores, which are positively correlated when maternal hemoglobin is below 110 g/L (139). Children whose mothers had a low dietary iron intake or low umbilical cord ferritin concentration during pregnancy have lower gross motor and fine motor scores than children whose mothers had a diet rich in protein and micronutrients (22, 140). The severity of impaired motor development is related to the timing and duration of ID. When ID occurs in the third trimester of pregnancy, the Peabody Developmental Motor Scales, Second Edition (PDMS-2) gross motor scores are lower (141). However, a study showed that prenatal ID or IDA were not associated with motor development in the offspring despite low umbilical serum ferritin concentration, possibly because iron supplementation was not considered during pregnancy (142).

### 6.2 Learning and memory

Prenatal IDA or low cord ferritin concentration is associated with impaired cognitive and intellectual development in offspring (22, 143–146). Memory is categorized into two distinct types: explicit memory, employed to recall past events, and implicit memory, related to motor and skill tasks and cognition (147). The explicit memory is assessed by electrophysiological measurements and behavioral memory performance, which include evoked imitation of immediate recall and delayed imitation of 1-week delayed recall. The study shows that prenatal ID can have a lasting effect on memory function in offspring, as evidenced by impaired recall and compromised encoding and retrieval processes (148). Fetuses with low cord ferritin or high ratio of porphyrin/heme zinc in the umbilical cord blood allocated more attentional resources to mother's voice and face recognition memory (24, 149, 150). Both the timing of the onset of ID and the age at which the infant's recognition memory is assessed influence the results (150). In the animal model, prenatal ID in rats impairs hippocampus-dependent trace fear conditioning and eye-blinking conditioning in offspring, indicating implicit memory is impaired (127, 128). The offspring of prenatal iron-deficient rats are more likely to rely on the striatum to navigate spatial memory tasks (126).

### 6.3 Affective and neurodevelopmental disorders

Accumulating evidence suggests that prenatal ID is associated with affective disorders in the offspring. In mice, prenatal ID reduces brain iron levels and increases susceptibility to anxiety- and depression-like behaviors in offspring (23). In rats, prenatal ID is associated with autism-like and schizophrenia-like behaviors in offspring, exhibiting abnormal pre-pulse inhibition of offspring's acoustic shock and sensitivity in novel environments (151). In humans, low maternal iron intake or ferritin level during pregnancy are related to an increased risk of autism in offspring (152, 153). In addition, offspring with low maternal hemoglobin concentration or anemia during pregnancy have an increased risk of schizophrenia, suggesting that maternal ID is a risk factor for schizophrenia in offspring (154).

## 7 Possible mechanisms underlying the neurological disorders caused by prenatal iron deficiency

### 7.1 Abnormal epigenetic modification

Epigenetic regulation refers to chemical modifications of DNA and histones that affect gene expression without altering the genetic code, such as DNA methylation, histone modification, regulation of non-coding RNA, and chromatin remodeling (155, 156). In animal models, maternal ID-induced dysregulation of gene expression in hippocampal neuronal development and functional pathways is related to aberrant DNA methylation (157). These pathways are the β-adrenergic signaling pathway, the CAMP-PKA signaling pathway, Rho GTPase signaling, and reelin signaling, all of which are involved in synaptogenesis and synaptic plasticity (157). In humans, lower levels of DNA methylation in the umbilical cord are related to lower maternal serum ferritin concentrations during the first trimester, and these relationships partially persist in children (158). The concentration of transferrin in pregnant women is associated with increased DNA methylation at cg09996156 (KIAA1324L), a regulator of the bone morphogenetic protein (BMP) pathway, which participates in apoptosis and autophagy and affects the development of the embryonic nervous system (158, 159). Iron is involved in two families of epigenetic modifications—Ten-Eleven Translocation (TET) proteins and Jumonji and AT-rich interaction domain-containing (JARID) proteins—both of which regulate gene expression during critical periods of brain development (160). The TET enzyme demethylates DNA by catalyzing the oxidation of 5-methylcytosine to form 5-hydroxymethylcytosine (5hmC), which serves as a stable epigenetic marker for neurons (161, 162). *Syt1* and *Nav2* are genes with high levels of 5hmC that play a role in neurogenesis and synaptic transmission (162, 163).

Histone modifications include methylation, acetylation, phosphorylation, and ubiquitination, with methylation and acetylation being the most common (164). The expression of JARID1B gene is downregulated in the hippocampus of the offspring of maternal iron-deficient rats (165) due to enrichment of histone deacetylase 1 (HDAC1) at the JARID1B promoter and the low acetylation level of H3K9 (166). Proteins containing JmjC domain are known as demethylases and can regulate transcription by removing methyl groups from lysine residues in the tail of histones (167, 168). Low JARID1B (Kdm5b) demethylation from trimethylated and dimethylated histone H3 lysine 4 (H3K4me1/2) leads to an increase in chromatin compaction and a decrease in transcriptional activity, whereas low JMJD3 (Kdm6b) and JHDM1d demethylates from H3K9me3 and H3K27me3 leads to a decrease in transcriptional repression of chromatin conformation (169–171). Therefore, alteration of JARID1B expression under ID can regulate transcription levels by altering chromatin structure, such as BDNF-related genes (165). Increased levels of H3K27me3 labeling are associated with promoter inhibition, and increased levels of H3K4me3 labeling are associated with promoter activity (172). Iron-deficient fetuses have an increased concentration of H3K27me3 and a decreased concentration of H3K4me3 in the hippocampus, which may be one of the mechanisms for decreased transcriptional activity of Bdnf-P4 (165, 173). Iron supplementation during the critical period of hippocampal development could partially increase JARID, but the recovery ability is limited (166). Choline supplementation during pregnancy reduces the expression of histone methyltransferase G9a and Suv39h1 in the hippocampus, which may be a potential mechanism for reversing maternal ID-induced HDAC1 enrichment and reduced H3K4me3 levels (173, 174).

MicroRNAs are non-coding single-stranded RNAs of approximately 22 nucleotides in length that are widely involved in the regulation of neurogenesis, development, apoptosis, cell differentiation, proliferation, and other biological processes by inhibiting the translation of messenger RNAs or promoting mRNA degradation (175). Prenatal ID alters miRNA expression in the brain, such as miR-200a and miR-200b, which may increase the risk of depression and anxiety-like behaviors in the offspring (23).

### 7.2 Mitochondrial dysfunction

Iron is involved in enzymes that make up the electron transport chain and the tricarboxylic acid cycle; therefore, it can influence brain development through energy metabolism (176). ID in hippocampal neurons alters the mRNA levels of genes related to mitochondrial function and energy metabolism, causing impaired mitochondrial respiration and glycolysis, which in turn affects the dendritic growth and branching (177). In the early stage of ID, only the oxidative capacity of mitochondria is affected, but in the later stage, the density of mitochondria is reduced, suggesting that there may be long-term effects on neurons (177). There are three main approaches in which chronic ID alters dendritic mitochondrial movement: first, an increase in the frequency of dendritic mitochondrial pauses decreases the speed of mitochondrial motion (178). On the one hand, a reduction in localized transient ATP may influence the ATPase activity utilized by motor proteins, such as dynein motor proteins and dynamins in transporting mitochondria (179, 180). On the other hand, ID may enhance the mRNA expression levels of blood–brain barrier and neuronal glucose transporters in the hippocampus (181). Extracellular glucose alters Milton GlcNAcylation, which regulates the mitochondrial motility by O-GlcNAc transferase (OGT) (182). Second, changes in mitochondrial fusion and fission gene expression in response to ID can reduce mitochondrial size by inhibiting OPA1-mediated fusion and stimulating DRP1-mediated fission (183, 184). Third, reduced anterograde mitochondrial movement and increased retrograde segmental velocity are observed in ID, while overall retrograde motion remains unchanged (178). Therefore, mitochondrial malfunction due to ID may contribute to long-term neurological damage and psychiatric disorders in offspring.

### 7.3 HPA axis dysfunction

Stress leads to activation of the hypothalamic–pituitary–adrenal (HPA) axis, which elevates glucocorticoid (GC) concentrations (185). Elevated GC leads to the apoptosis and atrophy of hippocampal neurons, which impairs neuroplasticity and leads to abnormal behavior (186). Glucocorticoid receptor (GR) in the hippocampus can regulate glucocorticoid levels through a negative feedback loop (187). GR binds to the cytoplasmic heat shock protein (Hsp) 40 and Hsp70 to form a GR–Hsp40/Hsp70 complex, which promotes GR folding and localization to the intermediate domain of Hsp90. The GR–Hsp90 complex alters the structure of the protein to allow it to bind GC (188–190). The binding of FK506-binding protein (FKBP51) and p23 to the GR–Hsp90 complex increases the binding affinity of GR to GC. After GC binds to the GR–Hsp90 complex, FKBP51 is replaced by FKBP52, which assists in nuclear translocation of the GC–GR heterocomplex and inhibits gene transcription of corticotropin-releasing hormone (CRH) in the nucleus (191, 192). On the one hand, when GC concentration is elevated, the increased expression of FKBP5 gene inhibits GR activity by limiting the translocation of the receptor complex to the nucleus (193). On the other hand, alterations in the self-phosphorylation state of GR regulate its transport from the cytoplasm to the nucleus (193, 194). The cognitive impairment of iron-deficient offspring may be related to the elevated serum glucocorticoid level and the reduced GR activity. In a mouse model of the offspring of iron-deficient mothers, serum glucocorticoid level is increased and GR activity is significantly reduced (195). In the case of ID in the brain, the hippocampus GC–GR signaling pathway can be inhibited by impaired GR–HSP90 complex formation and nuclear translocation, which affects the negative feedback regulation function of GR and hyperactivates the HPA axis (195). Therefore, HPA axis dysfunction due to prenatal ID may also be one of the important pathways for neurodevelopmental and behavioral abnormalities in offspring.

## 8 Future prospects

Although previous studies have shown associations between maternal obesity, diabetes, smoking, alcohol exposure, stress and fetal ID, the underlying mechanisms remain unclear. Identifying these factors associated with fetal ID can help prevent neuronal dysfunction-related diseases. Currently, because the gestation process in rodents is different from that of humans, they cannot provide adequate models for neurodevelopment, disorders, and mechanisms of ID in pregnant offspring. Rhesus monkeys with similar pregnancy cycles to humans are used as animal models for ID in pregnancy. In human cohort studies, most researchers have linked fetal ID to disorders such as cognitive impairment, autism, and schizophrenia. However, perhaps due to the different diagnostic criteria for fetal ID and cognitive function, studies have shown no association with cognitive function changes in adulthood (196–198). In addition, further research is needed to determine whether there is a clear link between fetal ID and ADHD.

Although it has been demonstrated that fetal ID affects the brain development of offspring, the relationship between neurological disorders and the specific molecular mechanism is still unclear. The study of maternal iron deficiency models will help us to further understand and lay a good foundation for treatment. Dietary therapy can enhance iron status in pregnant women at risk of or with mild ID during pregnancy. Pregnant women should eat more foods rich in ascorbic acid and carotenoids, such as kiwi fruit, and reduce their intake of foods that inhibit iron absorption, such as coffee, tea, and phytic acid in grains (199). In animal models, prenatal choline supplementation mitigated the expression of genes associated with ID-induced psychological disorders, such as schizophrenia, autism, and anxiety (200). Iron-deficient neurons treated with choline can stimulate dendritic growth, restore dendritic complexity, and improve ATP production rate and glycolysis but not be fully restored to normal (201). These changes are more pronounced in female rats (202). However, prenatal choline supplementation in iron-sufficient rats can dysregulate the expression of genes associated with cognitive and psychological disorders and promote epithelial to mesenchymal transformation by inhibiting fatty acid metabolism and oxidative phosphorylation activity, leading to cell adhesion and migration, which is similar to the adverse effects of ID (202). However, these changes do not affect the complexity of dendrites and the structural development of neurons (201). Prenatal choline supplementation attenuated ID-induced ADORA2 gene network in women and FEV gene network in men, which are associated with depression and attention disorders, respectively (202). In humans, existing studies indicate that choline supplementation during pregnancy may improve cognitive function in offspring (203, 204). In terms of the choline supplementation window during pregnancy, the cognitive ability improvement is lower in early pregnancy than in mid-pregnancy when choline intake is the same (204). Choline supplementation in late pregnancy enhances the attention maintenance ability of offspring (203). The recommended daily intake of choline for pregnant women is 450 mg/day (205). In the late stages of pregnancy, offspring with daily intakes of 930 mg/day demonstrated higher cognitive abilities than those with daily intakes of 480 mg/day (203). Additionally, maternal plasma choline levels were positively correlated with cognitive development in full-term infants (206). However, the dosage and duration of choline supplementation to alleviate the adverse reactions caused by ID during the prenatal period have not yet been conclusive. Moreover, in terms of human health, nearly 40%−50% of children with prenatal ID continue to experience intellectual disability and long-term neurological impairment despite iron supplementation (207). Early-life mitochondrial dysfunction is recognized as one of the potential factors for these psychiatric disorders (178). Supplementing dietary selenium can improve mitochondrial function by increasing the expression of selenoprotein K in the endoplasmic reticulum of neurons, promoting TfR-1 palmitoylation, and increasing intracellular iron levels (208). Idebenone is a Coenzyme Q10 analog that protects the mitochondria by acting as an antioxidant and increasing ATP production, thereby alleviating cognitive impairment. However, whether they can improve cognitive impairment in offspring of prenatal ID remains to be studied (209). Therefore, mitochondria are an attractive target for the design of alternative therapeutic interventions to prevent long-term neuropathology in many children, in addition to timely iron supplementation.

Various models (cells, organ tissues, and animals) will be utilized to rigorously validate whether target regulation can rescue or improve neurodevelopmental phenotypes through genetic manipulation (overexpression, knockout, and knockdown) and pharmacological intervention. Conducting standard reproductive toxicity and developmental toxicity studies in at least two animal species (typically one rodent and one non-rodent species such as non-human primates) to evaluate how different doses and administration timings (corresponding to various developmental stages) affect maternal and fetal health (including all organ systems, particularly the nervous and reproductive systems), as well as the long-term developmental outcomes of offspring. With strict regulatory and ethical oversight, the safety, tolerability, pharmacokinetic characteristics, and preliminary efficacy of fetal interventions in humans should be assessed, along with long-term postnatal follow-up. Professional societies should develop evidence-based clinical practice guidelines for fetal intervention procedures, clearly defining the indications, contraindications, operational standards, monitoring requirements, and long-term follow-up protocols. The following five aspects encompass ethical considerations regarding interventions during fetal development: the moral status of the fetus as a patient; the extreme uncertainty in risk-benefit assessments; the complexity of informed consent; equity, accessibility, and resource allocation; and the establishment of regulatory and oversight frameworks.
